# Harnessing ChatGPT for Thematic Analysis: Are We Ready?

**DOI:** 10.2196/54974

**Published:** 2024-05-31

**Authors:** V Vien Lee, Stephanie C C van der Lubbe, Lay Hoon Goh, Jose Maria Valderas

**Affiliations:** 1 Division of Family Medicine Yong Loo Lin School of Medicine National University of Singapore Singapore Singapore; 2 Department of Family Medicine National University Health System Singapore Singapore; 3 Centre for Research in Health Systems Performance National University of Singapore Singapore Singapore

**Keywords:** ChatGPT, thematic analysis, natural language processing, NLP, medical research, qualitative research, qualitative data, technology, viewpoint, efficiency

## Abstract

ChatGPT (OpenAI) is an advanced natural language processing tool with growing applications across various disciplines in medical research. Thematic analysis, a qualitative research method to identify and interpret patterns in data, is one application that stands to benefit from this technology. This viewpoint explores the use of ChatGPT in three core phases of thematic analysis within a medical context: (1) direct coding of transcripts, (2) generating themes from a predefined list of codes, and (3) preprocessing quotes for manuscript inclusion. Additionally, we explore the potential of ChatGPT to generate interview transcripts, which may be used for training purposes. We assess the strengths and limitations of using ChatGPT in these roles, highlighting areas where human intervention remains necessary. Overall, we argue that ChatGPT can function as a valuable tool during analysis, enhancing the efficiency of the thematic analysis and offering additional insights into the qualitative data. While ChatGPT may not adequately capture the full context of each participant, it can serve as an additional member of the analysis team, contributing to researcher triangulation through knowledge building and sensemaking.

## Introduction

### Background

**Figure 1:**
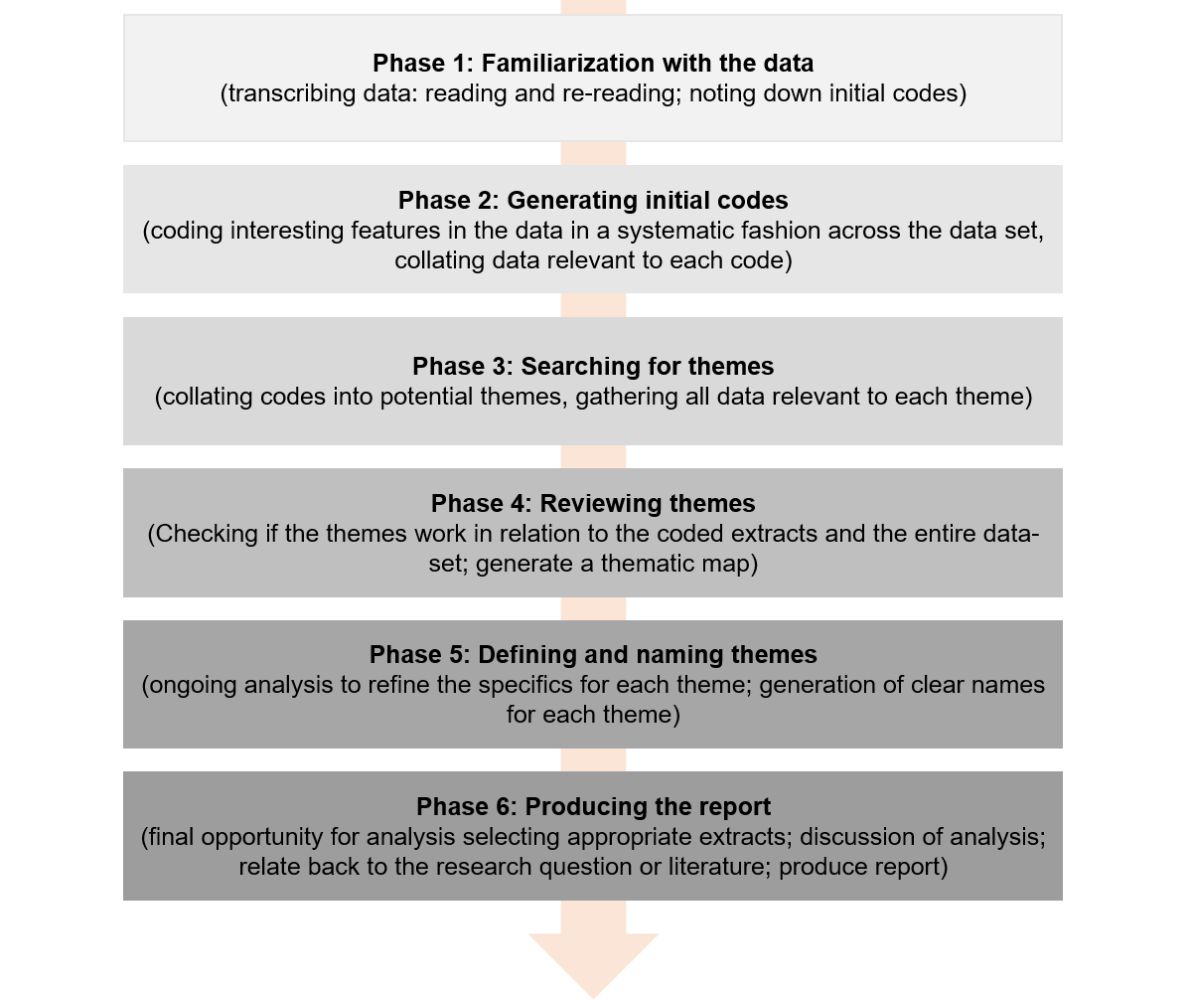
Braun and Clarke’s 6 phases of thematic analysis.

Thematic analysis is a method to analyze qualitative data, commonly obtained through semistructured interviews or focus groups, with the aim of identifying and interpreting patterns of meaning or themes within the data [1]. As a method, thematic analysis is inherently flexible and dependent on the researcher’s underlying philosophical assumptions [2]. For instance, positivist approaches may place greater emphasis on coding reliability, while interpretivist approaches may place more significance on reflexivity and the researcher’s role (including subjectivity) in knowledge production [2]. Accordingly, thematic analysis may be well-suited to meet varying research needs and requirements [3]. While there are multiple methods for thematic analysis, Braun and Clarke’s [1] 6 phases of thematic analysis is one of the most widely used approaches (Figure 1). The phases in Figure 1 were first defined in 2006, and the reflexive nature of their approach was further clarified in their 2019 publication [1,2].

Given the flexibility of thematic analysis, there is room for creativity when engaging with the data and exploring tools that may aid the researcher’s analytic process. With the increasing adoption of natural language processing (NLP) in health care research, such as diagnostic evaluation of electronic health records and the prediction of clinical outcomes based on consultation notes [4-6], researchers have begun to explore if there is space for artificial intelligence (AI) within the domain of qualitative research. To date, several AI-based tools, such as AILYZE (James Goh) and MonkeyLearn (Raul Garreta), are available to aid researchers in conducting thematic analysis [7,8]. For instance, AILYZE is able to summarize interview transcripts, provide suggestions for themes, and extract relevant quotes for each theme [7]. Nevertheless, full access to these tools often requires subscription payments, making them inaccessible for researchers and institutions that lack adequate financial resources.

In November 2022, OpenAI released version 3.5 of ChatGPT, a large language model-based chatbot capable of performing a wide range of text-based tasks based on context and past conversations (eg, summarizing research articles, answering domain-specific questions, and generating outlines for manuscripts) [9]. ChatGPT-3.5 is the chatbot adaptation of GPT-3.5 and is specifically optimized for back-and-forth conversations, though GPT-3.5 and ChatGPT-3.5 share the same foundational model. ChatGPT-3.5 is able to process a request and provide a response within a combined limit of 4096 tokens (ie, textual units, equivalent to approximately 3000 words in English), typically within a few seconds and free of charge [9,10].

Due to its electronic accessibility and free-to-use model, there has been a proliferation of discussion in the scientific community about incorporating GPT and ChatGPT into various aspects of research, including literature reviewing, data processing, and manuscript writing [11,12]. Qualitative studies have begun exploring the use of GPT and ChatGPT for conducting various aspects and types of qualitative analysis, from transcription cleaning to theme generation through thematic analysis [13-19]. Table 1 summarizes these studies by describing how GPT and ChatGPT were used in the analysis process, the main findings, and the challenges faced during the process. While all these studies are in preprint form and some are awaiting formal peer review, they provide an early glimpse into the feasibility of harnessing ChatGPT as an assistive tool when conducting qualitative analysis.

**Table 1:** Summary of studies that used ChatGPT or GPT for different phases of thematic analysis.

Reference	Country	GPT model	Use of ChatGPT or GPT	Findings	Challenges with ChatGPT or GPT
De Paoli (2023) [13]	United Kingdom	GPT-3.5 Turbo	The study used GPT to conduct thematic analysis, including generating initial codes, searching for themes, reviewing themes, and defining and naming themes.	GPT-3.5 Turbo was able to provide themes with synthetic descriptions. However, some inferred themes were not considered relevant by human researchers, and ChatGPT missed out on themes that were reported by human researchers.	Interviews had to be divided into chunks due to the token limit. Output is prompt-dependent (eg, asking for a different number of themes produced a different set of themes). Hallucination (eg, assigned incorrect code to theme).
De Paoli (2023, preprint) [14]	United Kingdom	GPT-3.5 Turbo	The study used GPT to build user personas (ie, fictional yet realistic descriptions of a typical or target user of a product [20]) based on interview transcripts using thematic analysis.	GPT-3.5 Turbo was able to generate 2 relevant personas based on challenges and needs identified during thematic analysis.	Biased toward creating specific types of user personas (eg, mostly middle-aged, from Italy) Required human intervention to refine codes and themes generated (eg, generated code with a truncated quote)
Gao et al (2023, preprint) [15]	Singapore and United States of America	GPT-3.5	The study explored the functionality of CollabCoder (a data management prototype incorporating GPT-3.5) in assisting with open coding, iterative discussions, and the development of codebooks.	Participants valued GPT-3.5 for reducing cognitive burden during coding, but some participants cited that summaries generated by GPT-3.5 are too detailed and not relevant.	Does not consider research questions or the intended direction of analysis if not explicitly instructed.
Mesec (2023, preprint) [16]	Slovenia	Information not available	The study used ChatGPT to conduct qualitative analysis using the grounded theory method.	ChatGPT was able to summarize and balance opposing ideas but tended to express ideas using descriptive terms at a lower level of abstraction compared to human researcher.	Hallucination (eg, made-up information in a summary of texts) Codes inadequately capture the content of the transcript Unproductive repetition of output Inappropriate use of terms (eg, “we can form some qualitative analyses”)
Tabone and de Winter (2023) [17]	Netherlands	GPT-3.5 Turbo and GPT-4-0613	The study used GPT to (1) conduct sentiment analysis, (2) provide meta-summaries of interviews, and (3) identify differences between 2 think-aloud transcripts.	Ratings (*r*=.98) and summaries generated with GPT-3.5 were strongly correlated or generally in line with those generated by human researchers. GPT-3.5 was also able to summarize the descriptive differences between 2 transcripts.	Prompt-dependent (eg, modified prompt increased correlation of ratings) Summary by GPT-4.0 was richer and touched on more facets than GPT-3.5 Turbo; however, some topics that were identified did not emerge in content analysis conducted by humans.
Taylor (2024) [18]	United States of America	Information not available	The study used ChatGPT to clean interview transcripts after using an artificial intelligence-assisted method to transcribe interviews.	ChatGPT cleaned redundant words and sentence fragments, but transcripts were more difficult to read due to ChatGPT connecting sentence fragments, which resulted in longer words per sentence.	Word and syntax errors remained in several transcriptions. Quality of transcription cleaning is dependent on the speech of the speaker (eg, clarity, pauses, and filler words). Limited input word count (500-600 words as of March 2023)
Xiao et al (2023, preprint) [19]	Canada and France	GPT-3	The study used GPT to conduct deductive coding using an expert-developed codebook.	GPT-3 achieved fair to substantial agreement with human researchers (Cohen 𝜅=0.38-0.61).	The model occasionally produced incorrect labels.

Whereas these previous papers have focused on the broader use of ChatGPT in thematic analysis, its integration into medical research has yet to be investigated. From this viewpoint, we therefore explore the use of ChatGPT for thematic analysis in the medical domain while addressing the unique challenges that arise within a medical context. We begin by assessing the use of ChatGPT for generating codes based on an interview transcript, followed by extracting themes from a list of generated codes. Subsequently, we use ChatGPT for tidying quotes for manuscript preparation. Finally, we use ChatGPT to generate interview transcripts, which may be used for various academic and educational purposes. For each application, we identify the areas where human intervention may still be required.

## Using ChatGPT in Thematic Analysis

Given ChatGPT’s ability to handle large textual data and provide sets of meaningful codes and themes, as demonstrated by De Paoli [13], ChatGPT has the potential to improve the efficiency of the thematic analysis process. As thematic analysis is typically conducted in a cyclical and iterative manner (eg, data collection and data analysis should occur concurrently, with insights from the analysis informing subsequent rounds of data collection and vice versa) [21], being able to digest and process large amounts of information efficiently (eg, by requesting a summary of an interview or generating an initial set of codes to help breakdown a transcript) can be helpful to researchers streamlining this cyclical process.

We used ChatGPT-3.5, which is free of charge, to illustrate the various ways ChatGPT can be used for thematic analysis. We have also used ChatGPT-4.0, which currently requires a fee, to see whether the results would improve when using a newer version of ChatGPT. Analyses were done on a transcript of the first episode of “Diabetes Discussion: A Diabetes UK Podcast,” in which 2 guests share their experiences of living with diabetes [22].

### Coding the Transcript

Our starting point is to investigate the capability of ChatGPT to code transcripts directly. To this end, we made the following request in ChatGPT:

The following is a transcript of an interview for a scientific paper focused on experiences of living with diabetes. Label the text by codes as is done in thematic analysis. Give the codes in the following format:

**CODE**

- First words of the sentence(s) that was/were labeled with this code

[transcript]

**Figure 2:**
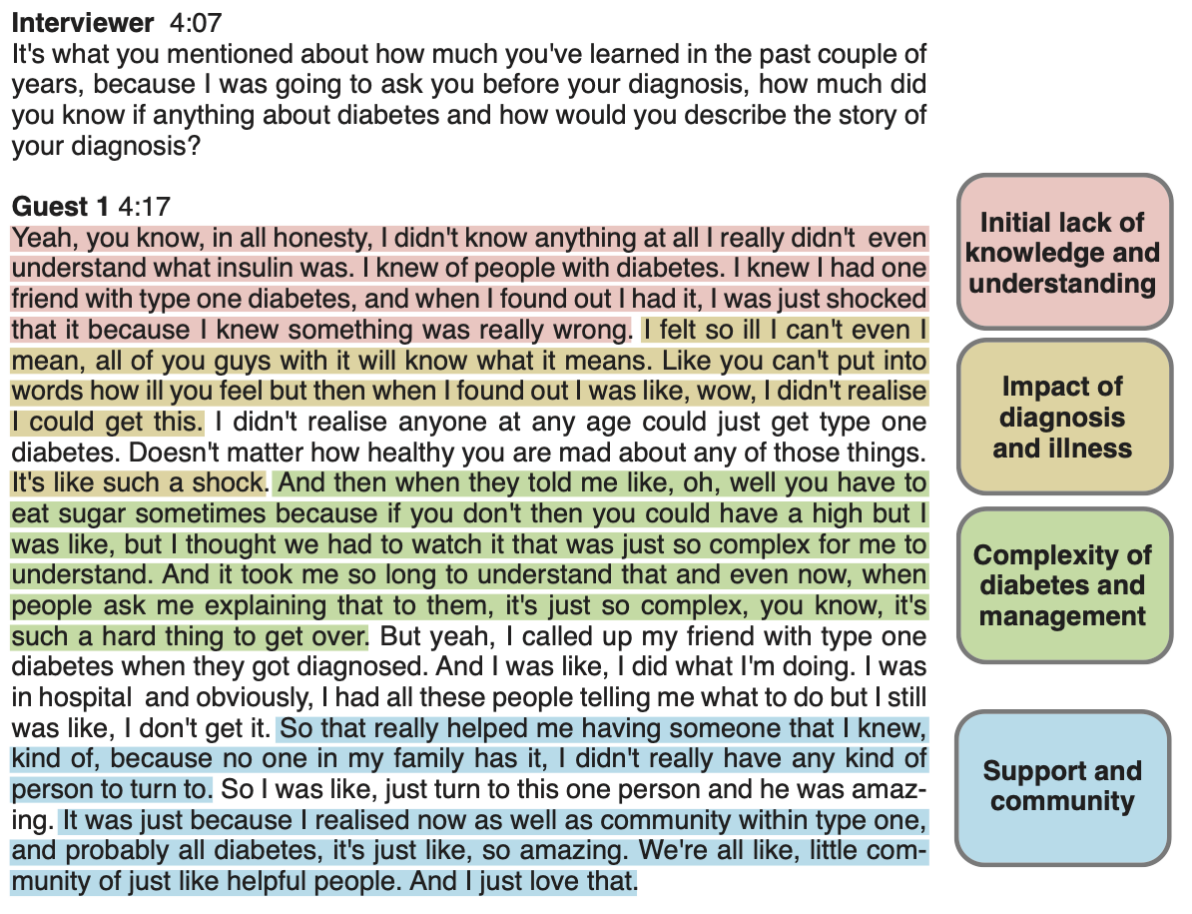
Transcript coded by ChatGPT-3.5. A larger portion of the coded transcript is given in Multimedia Appendix 1.

A section of the coded transcript by ChatGPT-3.5 is displayed in Figure 2. ChatGPT-3.5 successfully identified multiple codes in the transcript within a single answer, and the corresponding codes match the textual content. However, as the transcript progresses (Multimedia Appendix 1), the coding by ChatGPT-3.5 becomes less detailed. While the subsequent answer contains multiple topics, ChatGPT-3.5 only assigned a single code, resulting in a loss of information in the coded output. Additionally, the second interview question (Multimedia Appendix 1) is also coded, which is not a common practice in thematic analysis.

Using ChatGPT-4.0 for the same analysis results in a noticeable improvement in the output (Figure S2 in Multimedia Appendix 1). Not only does ChatGPT-4.0 ignore the transcript of the sections corresponding to the interviewer, but it also captures more details of the transcript. ChatGPT-4.0 assigned 5 different codes for the second question (as opposed to one assigned by ChatGPT-3.5), resulting in a set of codes that give a more complete picture of the transcript.

ChatGPT demonstrates a promising ability to code transcripts, but its performance depends on the GPT model used. Consequently, it is still necessary for a human researcher to review the codes generated and ensure that the codes appropriately capture all essential data. Furthermore, while the codes generated by ChatGPT sufficiently describe important concepts within the transcript, initial codes generated by human researchers during data analysis may continue to evolve to become more specific or reworded to better capture the data as new transcripts are analyzed. Given ChatGPT’s limited context window, ChatGPT may not remember the full text of an interview transcript or the codes it previously generated, resulting in a loss of contextual understanding. For these reasons, a human researcher will be required to consolidate the codes generated to ensure that the codes adequately capture concepts or patterns of interest within the context of the whole data set.

### Extracting Themes From Codes

Another way that ChatGPT may be used is to extract the themes and subthemes from the generated codes. These codes may be obtained either from ChatGPT or a human analyzer. We used the following request:

The following codes were obtained via coding of a transcript. Please identify the overarching themes and subthemes as is done in thematic analysis. These themes should have as little overlap as possible, and will be used in a scientific paper focused on experiences of living with diabetes. Use the following format:

**THEME**

*Subtheme*:

- codes that belong to this subtheme

*Subtheme*:

- codes

**THEME**

*Subtheme*

...

[list of codes]

**Figure 3:**
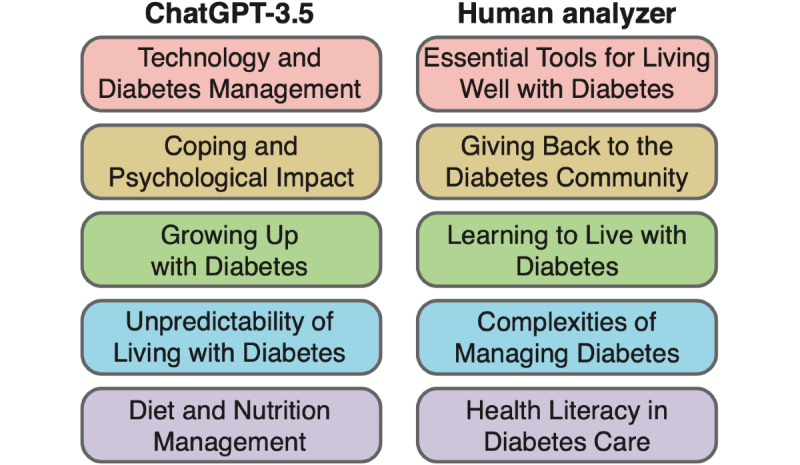
Themes identified by ChatGPT-3.5 (left) and the human analyzer (right) from 81 codes.

The resulting themes are shown in Figure 3; the subthemes are tabulated in Table S1 in Multimedia Appendix 1. The themes and subthemes were derived from 81 codes obtained through the coding of the Spotify (Daniel Ek and Martin Lorentzon) transcript. Both ChatGPT and the human analyzer identified 5 unique themes, though their analyses had notable differences. For example, ChatGPT identified the “diet and nutrition management” theme, a subject that the human analyst neither classified as a theme nor a subtheme (Table S1 in Multimedia Appendix 1).

We conducted several rounds of analysis with ChatGPT-3.5 by resubmitting the same prompt, which led to interesting variations in the identified themes. For example, in the second analysis round, ChatGPT identified the “pregnancy and diabetes” theme (Table S1 in Multimedia Appendix 1), whereas pregnancy only emerged as a subtheme in ChatGPT’s first analysis round. The human analyzer, in contrast, did not identify pregnancy as a theme or a subtheme in their analysis.

It is impossible to state which of these themes more accurately reflects the essence of the interview, as there is no absolute truth in thematic analysis. Instead, we see the identification of different themes as an advantage. After all, a greater diversity in themes indicates that the codes were interpreted from different angles, thereby adding more layers to the overall analysis. In this framework, ChatGPT should be viewed as an additional team member when doing thematic analysis by offering fresh perspectives and proposing alternative interpretations of the identified codes.

Similar to the coding process, all themes and subthemes generated by ChatGPT should still be reviewed by human researchers to ensure that the themes and subthemes generated are aligned with the research question or questions and that essential data have been appropriately captured by the themes.

### Tidying Up Quotes

While quotes in manuscripts are presented verbatim as much as possible, qualitative researchers often face the need to balance succinctness due to word count limits while maintaining the faithfulness of the quote to the participant’s intended meaning. Examples of this include using ellipses to indicate that a portion of the quote (irrelevant to the point being made) has been cut, correction of words to maintain the grammatical integrity of the sentence, or removal of filler words such as “ah” or “um” [23]. We explored the potential use of ChatGPT to tidy up quotes from the interview transcript for manuscript preparation using the following request:

Clean the following transcript so it may be used as a direct quote for an academic paper:

[quote]

Omit all text that is not essential for the main message. Any altered or inserted words must be shown between square brackets, “[]”, and omitted text must be replaced by three dots, “…”.

**Figure 4:**
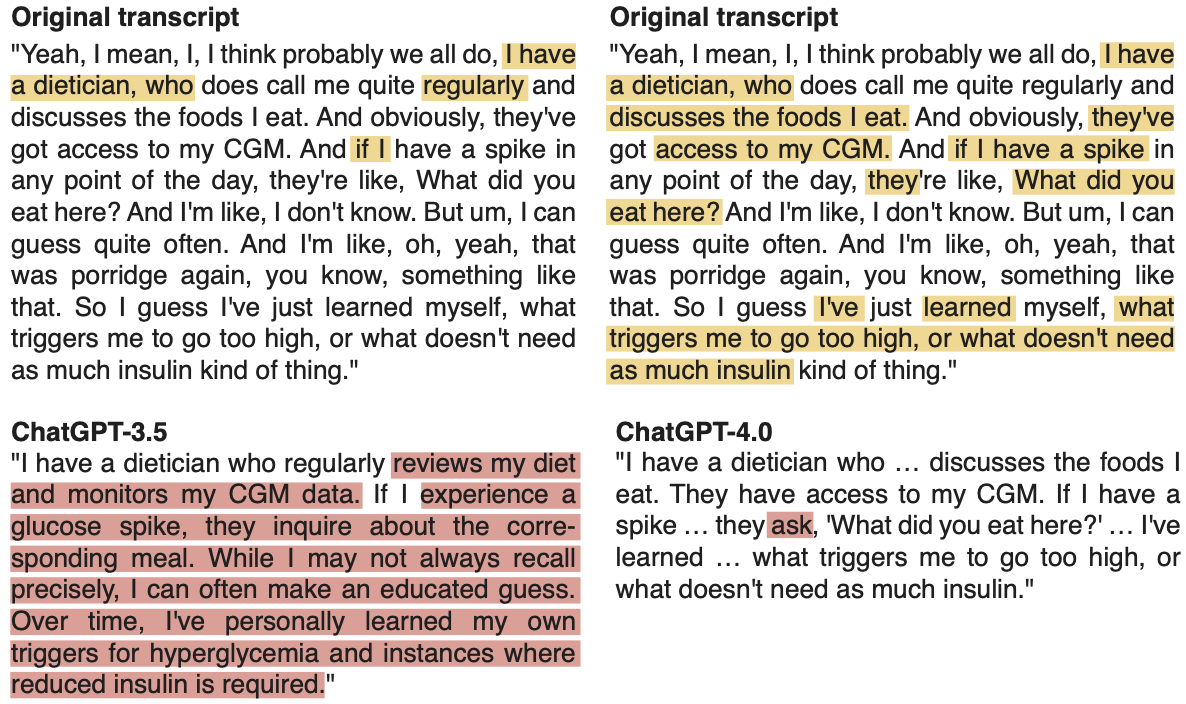
Direct quotes generated by ChatGPT-3.5 (left) and ChatGPT-4.0 (right). The yellow highlights indicate the text that has been included in the quote, whereas the red highlights indicate the text that has been rephrased by ChatGPT without proper use of square brackets.

As shown in Figure 4, ChatGPT-3.5 struggled to tidy up the quote for manuscript writing purposes. A substantial portion of the text underwent revision without proper use of square brackets, despite our explicit request to denote any modifications or insertions. Additionally, sections of the text were interpreted by ChatGPT-3.5, which deviated from our initial instructions. In contrast, ChatGPT-4.0 performed the task more effectively (Figure 4). The generated quote contains only one word that should have been added between square brackets, while the remainder of the quote is indeed correctly extracted from the original transcript. Nevertheless, depending on how the quote is used to describe the data, one may argue that too much text has been omitted from the original transcript, filtering out the more personal viewpoints or emotional content from the final quote. Accordingly, quotes tidied up by ChatGPT should be reviewed by human researchers to determine if the quotes provide sufficient information and context to further elaborate on the theme or subtheme discussed. As tidying up quotes during manuscript preparation is less time-consuming than coding and theme generation, researchers should consider if the steps needed to generate a tidy quote outweigh the manual process.

### Creating Transcripts

Finally, we have explored the potential of ChatGPT to generate transcripts of interviews. To this end, we made the following request to ChatGPT:

Generate a transcript of an interview for a scientific study with a diabetes patient about their experience with living with diabetes. Required length: ~1500 words. Use the following format:

Interviewer mm:ss

[Question 1]

Patient 1 mm:ss

[Answer]

Include stop words, pauses, and words such as “I think..,”, “uhh”, and “I mean...”, so it looks like an authentic transcript.

**Figure 5:**
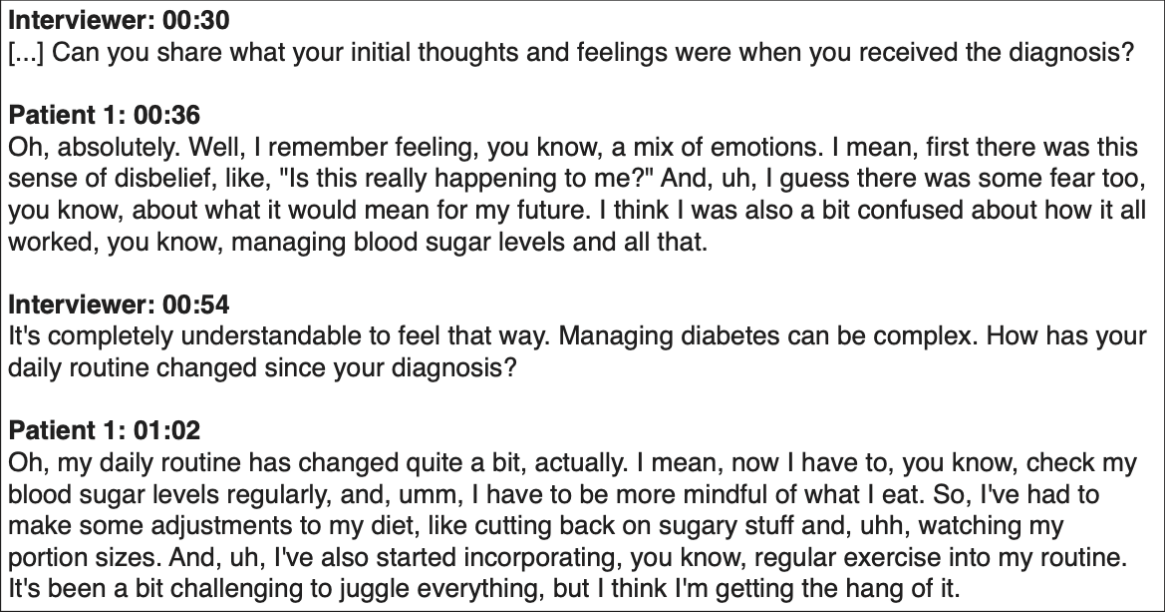
Snapshot of the transcript generated by ChatGPT-3.5. The full transcript is given in Multimedia Appendix 1.

This request was made without any additional instructions, such as an example transcript or a list of themes or codes. A snapshot of the generated transcript is shown in Figure 5. The resulting transcript matches the requested format and uses the stopwords that were asked for, making it similar to an actual conversation between 2 people. However, some differences exist between the generated transcripts and those from real-life interviews. The ChatGPT-generated transcript has a very direct question-answer structure, with all answers being on-point and of similar length. In contrast, a real-life interview is often more organic. For example, the interviewee may not understand the question and give answers of various lengths, and the interviewer may go in more depth before moving on to the next question.

Despite these differences, the ChatGPT-generated transcripts have multiple advantages. By slightly adjusting the ChatGPT request, the generated transcripts may focus on any topic of interest or feature different respondent characteristics. This adaptability of ChatGPT allows for the quick generation of a large and diverse collection of transcripts. Furthermore, generating transcripts with ChatGPT eliminates any privacy and confidentiality concerns, which is a common issue with actual interviews.

Given these advantages, we envision several potential applications for the ChatGPT-generated transcripts. First, these transcripts may be used as instruction material for students learning thematic analysis. A second approach worth investigating is to use the generated transcripts as a training set for NLP models, particularly in topic modeling. As real-life transcripts are often limited or hard to get, ChatGPT offers a practical way to expand the data set, thereby exposing the model to a larger diversity of text. Further research into the feasibility of using generated transcripts as training data for NLP models is essential, taking into account the quality, diversity, and representativeness of the generated transcripts and the potential influence of the training set on the model’s performance. It will be crucial to maintain a strict separation between training (ie, generated) data and real transcripts to ensure that any insights are obtained exclusively from real transcripts.

In short, while ChatGPT-generated transcripts cannot (yet) replicate the nuanced complexities of real interactions, they may be a promising source of training data for various academic and educational applications. Unfortunately, these generated transcripts may also open the opportunity for data falsification and be claimed as collected research data, especially since real data may be costly and time-consuming to obtain. To preserve the integrity of research, it is essential to ensure complete transparency regarding the origins of transcripts and their specific use within the analytical framework.

## Challenges When Using ChatGPT for Thematic Analysis

While ChatGPT is a promising assistive tool for thematic analysis, previous studies have identified challenges when working with ChatGPT (Table 1) [13-19]. Major challenges relevant to thematic analysis include hallucination (ie, responses produced by the system that are not justified by the data used), the output being prompt-dependent (eg, prompts requesting the same output but phrased differently will lead to different outputs), and missing themes or codes previously reported by researchers [13]. Similarly, we encountered several challenges when using ChatGPT to conduct thematic analysis. When working with patient data, the primary concern is data confidentiality. Inputs to ChatGPT may be used as training data to improve their services, and network activities may be shared with third parties [24]. For this reason, uploading sensitive information, such as patient interview transcripts, to ChatGPT should be avoided. This precaution restricts the use of ChatGPT for coding (ie, phase 2 in Figure 1) unless the transcript holds no confidential information.

A more practical challenge is that ChatGPT has a word limit for each prompt, which may prevent users from inputting full transcripts and very long lists of codes or quotes. One potential solution is to split the input. However, as discussed above, ChatGPT has a limited context window, so it may forget the earlier parts of the interview transcript and/or codes it previously generated. As a result, ChatGPT may not be able to adequately capture patterns of ideas or concepts within the context of the whole data set. When coding, researchers consider existing knowledge (eg, the research question or current information about the topic), knowledge obtained through data collection (eg, interviews and field notes), and existing codes from previously analyzed transcripts. Without further information beyond the input, ChatGPT may adopt a narrower lens and generate results that are highly specific to a singular transcript. Accordingly, at this point in time, it is still essential for human researchers to collate the codes generated for each transcript and review them within the context of the study.

In the context of understanding text, ChatGPT, though advanced, may not capture every nuance that a human analyst would pick up [25]. In certain instances, ChatGPT may overlook underlying emotions or implicit themes that would otherwise be evident to human analysts. It is thus important to review the output of ChatGPT to ensure that all essential aspects of the transcript are captured in its thematic analysis. Without taking into account the context of participants (eg, gender, age, and culture), there is concern that the experiences shared by participants may be diluted when analyzed by ChatGPT. As such, output from ChatGPT should be recognized as complementary rather than a replacement for analysis conducted by human researchers. Beyond that, we also found that ChatGPT sometimes excludes existing codes or introduces new codes when generating themes. Hence, it is advisable to double-check whether all codes have been correctly assigned to the identified themes and subthemes.

Finally, ChatGPT may give different answers to the same questions, leading to nonreproducible results. Yet, in the context of thematic analysis, we do not see this variability as a drawback, as humans would also generate different results when doing thematic analysis. Instead, the different responses from ChatGPT may be seen as an opportunity because they may provide new insights that were not captured during the first round of thematic analysis.

## Conclusions and Recommendations

**Figure 6:**
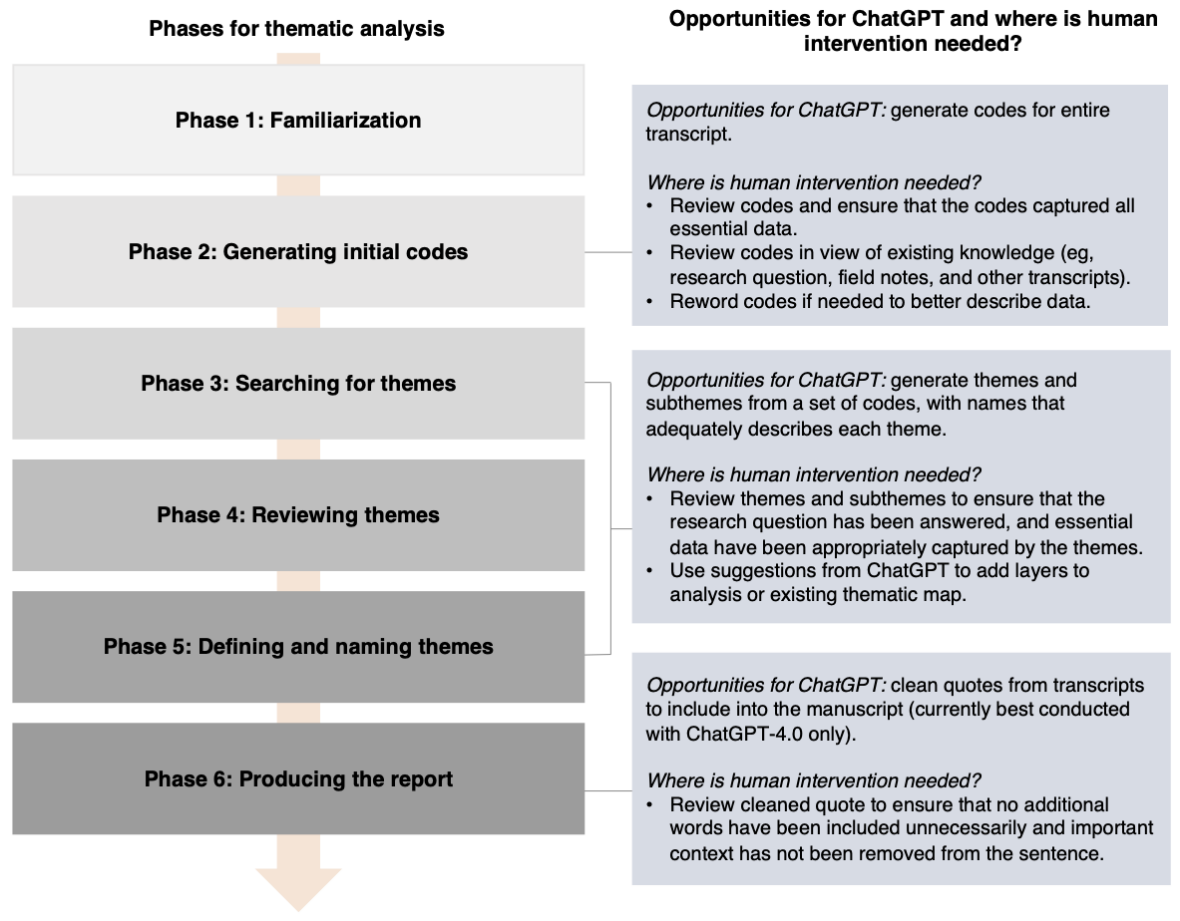
Opportunities for the usage of ChatGPT during the thematic analysis process and where human intervention is needed.

ChatGPT has the potential to enrich the field of qualitative research. In our investigation, ChatGPT demonstrated its ability to code interview transcripts, generate themes from a list of codes, tidy quotes for manuscript preparation, and generate unique transcripts for education and training purposes. Nevertheless, limitations such as the inability to manage multiple transcripts and not fully capturing nuanced data essential to the research question necessitate the involvement of human researchers to collate and review the output generated. At this stage, ChatGPT requires human-AI collaboration, where researchers have to remain in the loop to intervene when necessary [13,15]. We present Figure 6 to show the opportunities available for ChatGPT to assist in thematic analysis and areas where human involvement is still required.

Given the need for considerable interaction between ChatGPT and human researchers, it will be more valuable to recognize ChatGPT as an additional member of the analysis team, contributing to researcher triangulation by adding to knowledge building and sensemaking, rather than a replacement for human researchers. However, with the ongoing progress in the field of natural language processing, the role of ChatGPT in qualitative research will evolve rapidly. This fast-paced development, in combination with the growing use of ChatGPT in research, necessitates further discussions regarding the use of ChatGPT in qualitative research. For example, how should ChatGPT’s contribution be acknowledged, and what are the best practices regarding prompt formulation? Another important point of consideration is the confidentiality of the data, especially when working with patient data such as interview transcripts. The recent ChatGPT data breach in March 2023 should encourage researchers to remain mindful of the implications when working with AI tools that store data, regardless of purpose [26].

In summary, ChatGPT has the potential to function as a valuable tool during analysis, enhancing the efficiency of the thematic analysis and offering additional insights into the qualitative data. While this viewpoint remains an exercise to investigate the potential feasibility of using ChatGPT for thematic analysis, findings from the investigation can serve as a starting point for future studies that intend to further push the boundaries of AI involvement within qualitative research.

## Appendix

Multimedia Appendix 1 Transcripts and table.

## Abbreviations

AI: artificial intelligence
NLP: natural language processing
