# Addendum to "Harnessing ChatGPT for Thematic Analysis: Are We Ready?"

**DOI:** 10.2196/68963

**Published:** 2025-06-11

**Authors:** Stephanie C C van der Lubbe, V Vien Lee, Lay Hoon Goh, Jose Maria Valderas

**Affiliations:** 1 Division of Family Medicine Yong Loo Lin School of Medicine National University of Singapore Singapore Singapore; 2 Department of Family Medicine National University Health System Singapore Singapore; 3 Centre for Research in Health Systems Performance National University of Singapore Singapore Singapore

We recently published our viewpoint titled “Harnessing ChatGPT for Thematic Analysis: Are We Ready?” [1], in which we explored the potential of using ChatGPT-3.5 and ChatGPT-4 (OpenAI) to perform thematic analysis. Our viewpoint highlighted the strengths and limitations of this approach, considering factors like confidentiality and interpretive depth. Since our publication, OpenAI released a new series of reasoning models–the o-series–with the o1-preview launched in September 2024 and the o3 model released in April 2025. These models offer enhanced reasoning capabilities suitable for complex tasks [2], and present new opportunities for leveraging ChatGPT in thematic analysis.We reanalyzed the full “Diabetes Discussion: A Diabetes UK Podcast” transcript [3] (used in our original paper) using ChatGPT-o1-preview, yielding new insights that form the basis of this addendum.

With the context window increased from 8,192 to up to 200,000 tokens compared to ChatGPT-4 [4], the new o-series mark a substantial leap forward in thematic analysis capabilities. Previously, we were limited to uploading transcript segments, and context was partially lost between analysis steps [1]. Now, entire transcripts can be uploaded, and multiple thematic analysis steps can be conducted within a single, continuous conversation. These improvements enable a more cohesive, in-depth analysis, with contextual understanding maintained throughout the process. Specifically, this allows for more precise code and theme assignments by maintaining a clear link between the codes and the original transcript.

A potential workflow leveraging these upgrades might be as follows: first, initial codes are generated from the transcript attached to the prompt. Next, closely related codes are combined to form a structured set of overarching codes with corresponding subcodes by prompting ChatGPT with tailored instructions. Subsequently, the same prompt as in our original paper can generate themes and subthemes based on these codes. Since the entire process occurs within a single conversation, context is continuously retained, provided it does not exceed the context window. Additionally, while previous versions “hallucinated” codes and subcodes, ChatGPT-o1 demonstrated enhanced accuracy, generating themes and codes without any hallucination during our test runs.

Some words of caution are warranted. Although the new o-models have significantly advanced beyond versions 3.5 and 4, they should still be seen as an additional member of the analysis team rather than a replacement, with outputs needing to be verified and complemented by human researchers. Furthermore, prompt engineering is essential. For example, the resulting codes lacked clear organizational structure, with codes appearing out of sequence relative to the original transcript. Consequently, detailed instructions on coding and output presentation are required. Moreover, the new o-series' ability to analyze larger volumes of text inherently increases the number of generated codes and quotes. Researchers should, therefore, establish workflows to ensure clear and efficient management of outputs. We recommend using the Generative Pre-trained Transformers (GPT) Application Programming Interface (API), which supports structured data input and enables customized outputs in formats (eg, CSV) optimized for data management.

In summary, the new o-series offer a powerful tool with enhanced contextual understanding for more efficient and in-depth thematic analysis. With these rapid developments in less than a year, it is exciting to consider how ChatGPT’s role in qualitative research will continue to evolve, with improvements in context, accuracy, and analytical capabilities on the horizon.
