# Supplementary material for: Harnessing ChatGPT for Thematic Analysis: Are We Ready?
Source: J Med Internet Res. 2024 May 31;26:e54974. doi: 10.2196/54974 (PMC11179012; doi:10.2196/54974)
Supplement: Multimedia Appendix 1 [file jmir_v26i1e54974_app1.docx]

**Multimedia Appendix 1**

**Harnessing ChatGPT for thematic analysis: Are we ready?**

V Vien Lee^[1,#]^, PhD; Stephanie C. C. van der Lubbe^[1,#]^, PhD; Lay Hoon Goh^[1, 2]^, MBBS; Jose Maria Valderas^[1,2,3]^, MD, PhD

^[1]^ Division of Family Medicine, Yong Loo Lin School of Medicine, National University of Singapore, Singapore, Singapore

^[2]^ Department of Family Medicine, National University Health System, Singapore, Singapore

^[3]^ Centre for Research in Health Systems Performance, National University of Singapore, Singapore, Singapore

^[#]^ Authors contributed equally to this work.


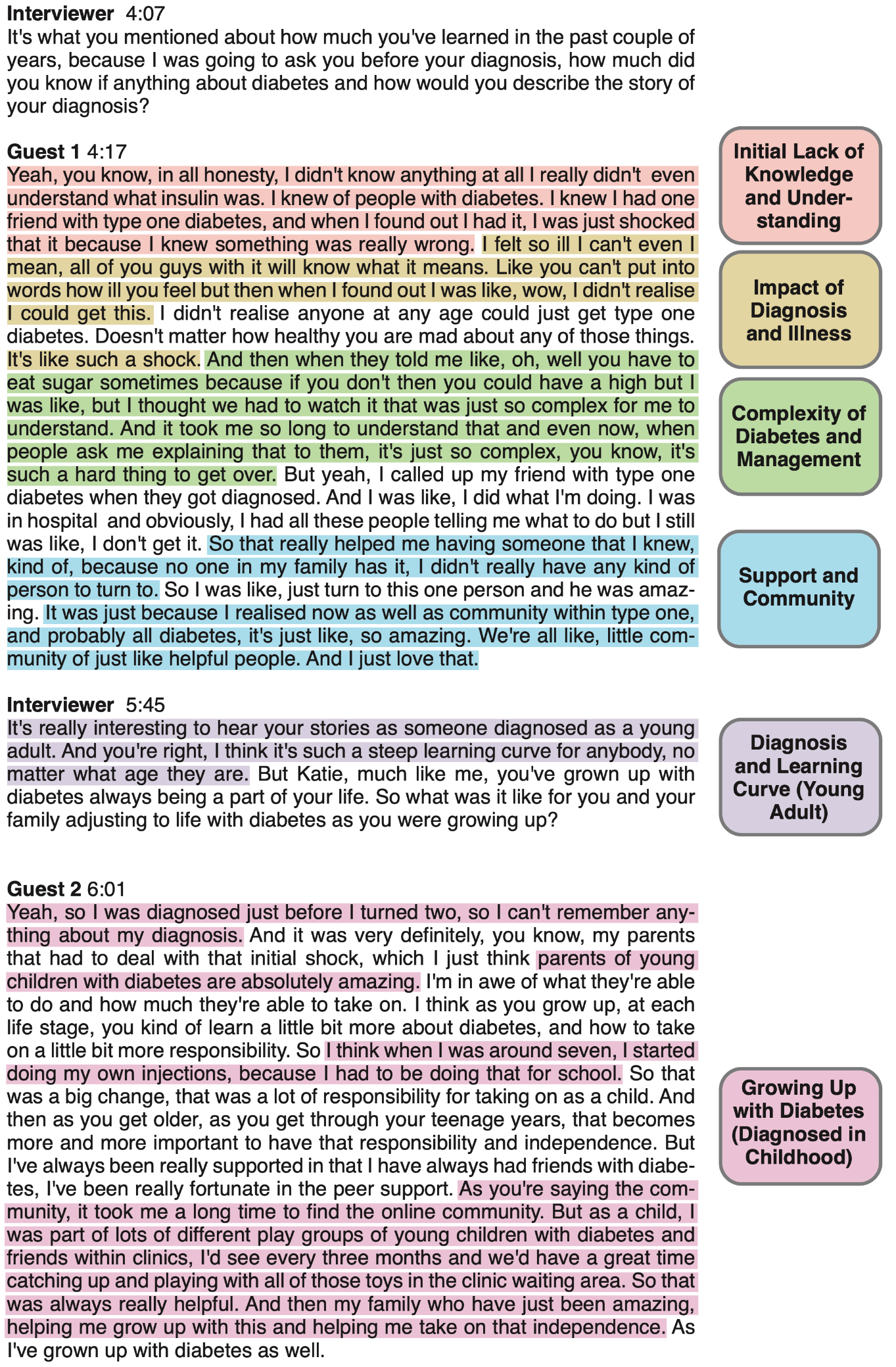


**Figure S1**. Transcript coded by ChatGPT-3.5.


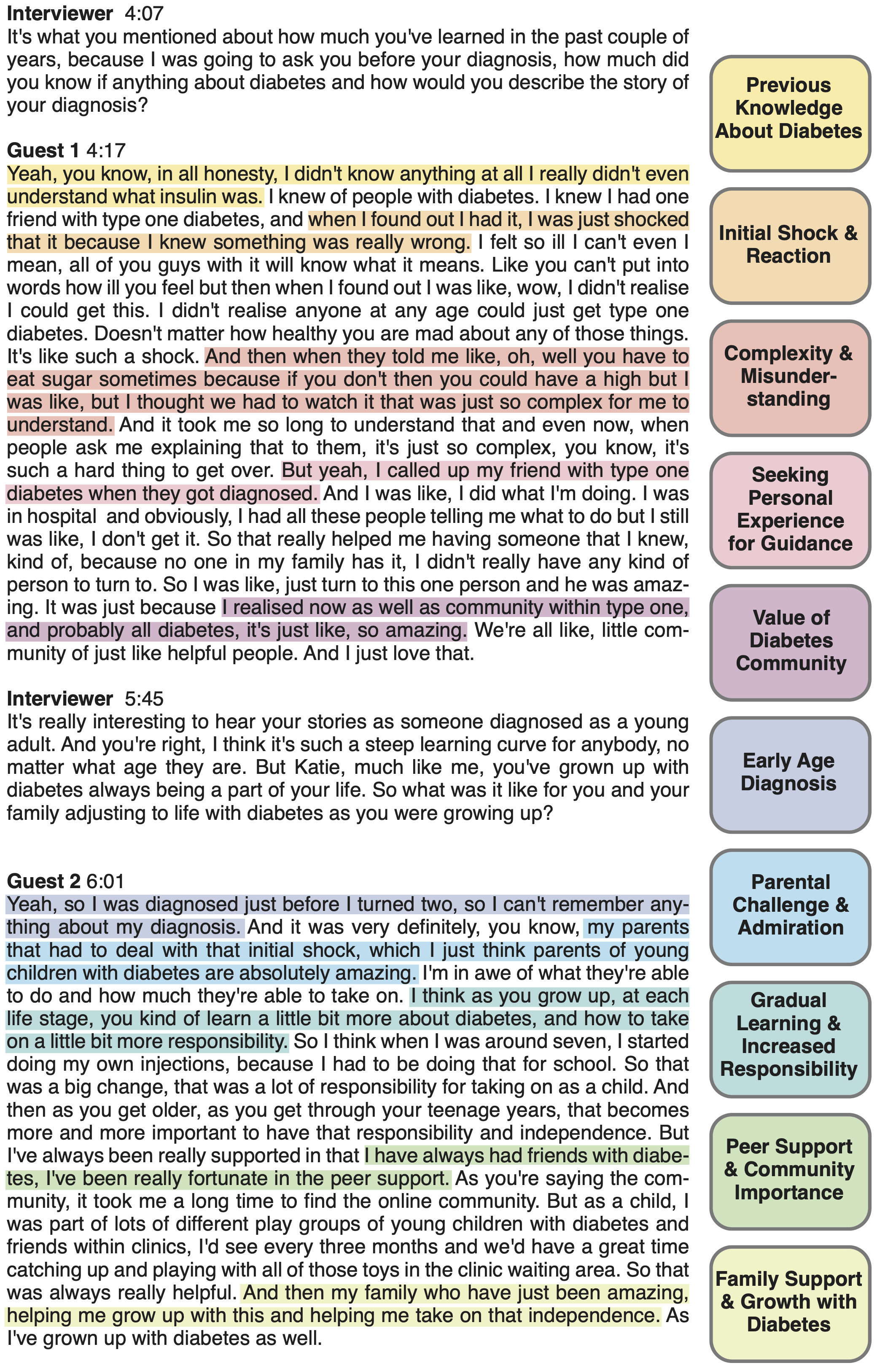


**Figure S2**. Transcript coded by ChatGPT-4.0.

**Transcript generated by ChatGPT-3.5**

**Interviewer: 00:00**

Good morning. Thank you for participating in this interview for our scientific study on the experience of living with diabetes. Let's start with a basic question: Can you tell me when you were diagnosed with diabetes and what type of diabetes you have?

**Patient 1: 00:12**

Sure, uhh, I was diagnosed about five years ago. It was, umm, I think it was in my early 40s. I have type 2 diabetes. Yeah, I mean, it was a bit of a shock, you know? I didn't really see it coming.

**Interviewer: 00:30**

I can imagine that was quite surprising. Can you share what your initial thoughts and feelings were when you received the diagnosis?

**Patient 1: 00:36**

Oh, absolutely. Well, I remember feeling, you know, a mix of emotions. I mean, first there was this sense of disbelief, like, "Is this really happening to me?" And, uh, I guess there was some fear too, you know, about what it would mean for my future. I think I was also a bit confused about how it all worked, you know, managing blood sugar levels and all that.

**Interviewer: 00:54**

It's completely understandable to feel that way. Managing diabetes can be complex. How has your daily routine changed since your diagnosis?

**Patient 1: 01:02**

Oh, my daily routine has changed quite a bit, actually. I mean, now I have to, you know, check my blood sugar levels regularly, and, umm, I have to be more mindful of what I eat. So, I've had to make some adjustments to my diet, like cutting back on sugary stuff and, uhh, watching my portion sizes. And, uh, I've also started incorporating, you know, regular exercise into my routine. It's been a bit challenging to juggle everything, but I think I'm getting the hang of it.

**Interviewer: 01:25**

It sounds like you've made some positive changes to manage your diabetes. Can you tell me about any specific challenges you've faced in your daily life as a result of living with diabetes?

**Patient 1: 01:34**

Oh, definitely. I mean, there have been some challenges for sure. Umm, one thing that comes to mind is, you know, eating out or social gatherings. It can be a bit awkward sometimes, trying to make healthy choices while also, you know, not making a big deal out of it. And, uh, I've had my moments of frustration too, especially when my blood sugar levels, like, they just don't seem to cooperate, no matter what I do. It's a bit frustrating, I mean, you know, I'm trying my best here.

**Interviewer: 01:56**

I can see how those situations can be tough. How about the emotional aspect of living with diabetes? Have you experienced any emotional ups and downs related to your condition?

**Patient 1: 02:06**

Oh, definitely. I mean, there have been times when I've felt a bit down about it. You know, I think it's natural to have those moments of, like, "Why me?" or feeling, you know, a bit overwhelmed. And then there are moments when I'm proud of myself, you know, when I've managed to keep my blood sugar levels in check or I've hit my exercise goals. It's a bit of a rollercoaster, I mean, emotionally.

**Interviewer: 02:25**

Absolutely, it's important to acknowledge both the challenges and the victories. How has your support system been throughout this journey? Have you received support from family, friends, or healthcare professionals?

**Patient 1: 02:35**

Yeah, I'm really grateful for the support I've received. Umm, my family has been, you know, quite understanding and they've tried to make, like, healthier meals when I'm around. And my friends, well, they've been pretty great too, you know, making sure there are options that I can eat if we go out. And my doctor, uh, they've been really helpful in explaining things to me and, you know, answering my questions. So, yeah, I think having a good support system makes a big difference.

**Interviewer: 02:57**

It sounds like you have a strong network to lean on. Looking back, what advice would you give to someone who has recently been diagnosed with diabetes?

**Patient 1: 03:05**

I would say, you know, take it one step at a time. It can be overwhelming at first, but you'll learn as you go. And, uh, don't be afraid to ask questions, whether it's from your doctor or, you know, support groups online. And, umm, also, I think it's important to be kind to yourself. There will be days when things don't go as planned, and that's okay. You're doing your best, and that's what matters.

**Interviewer: 03:23**

Great advice. Finally, can you share any positive aspects that have come from your experience with diabetes? Have there been any unexpected silver linings?

**Patient 1: 03:32**

Oh, yeah, definitely. I mean, I think, you know, having diabetes has made me more conscious of my overall health. I've, umm, I've learned a lot about nutrition and exercise that I might not have paid as much attention to before. And, you know, it's also brought me closer to some people in my life. Like, my friends and family, they've really shown their support and it's been, you know, heartwarming. So, yeah, I think there have been some positive aspects despite the challenges.

**Interviewer: 03:54**

That's wonderful to hear. Thank you so much for sharing your experiences with us today. Your insights will certainly contribute to our understanding of living with diabetes.

**Patient 1: 04:02**

You're welcome. I'm glad I could help. If my experiences can, you know, help someone else going through the same thing, then that's great. Uhh, thanks for having me.

**Interviewer: 04:10**

Thank you again, and take care.

| **ChatGPT­-3.5: Analysis 1** | | **ChatGPT­-3.5: Analysis 2** | | **Human analyzer** | |
| --- | --- | --- | --- | --- | --- |
| **Themes** | **Subthemes** | **Themes** | **Subthemes** | **Themes** | **Subthemes** |
| Technology and Diabetes Management | - Use of Diabetes Technology - Healthcare Support and Education | Diabetes Management Strategies | - Technological Aids - Diabetes Education and Peer Support - Dietary Management - Emotional and Mental Aspects | Essential Tools for Living Well with Diabetes | - Preparation and Planning - Supportive Network and healthcare Team - Continuous Self-learning - Access to Digital Health Tool |
| Coping and Psychological Impact | - Psychological and Emotional Struggles - Stigma and Normalization | Stigma and Normalization | - Battling Stigma - Advocating for Normalcy | Giving Back to the Diabetes Community | - Tackling Stigma and Normalizing Diabetes |
| Growing Up with Diabetes | - Diagnosis at Young Age - Independence and Responsibility - Impact on Social Life and Education | Impact of Diabetes Diagnosis on Life | - Shock and Lack of Knowledge - Supportive Environment and Education - Becoming Independent with Age - Impact on Relationships | Learning to Live with Diabetes | - Parent's Role in child's diagnosis - Able to successfully adapt to living with diabetes |
| Unpredictability of Living with Diabetes | - Unpredictability of Diabetes Management - Challenges in Pregnancy with Diabetes - Diagnosis and Initial Reactions - Support Networks and Community | Unpredictability of Living with Diabetes | - Unpredictability of Diabetes Management - Navigating Uncertainties | Complexities of Managing Diabetes | - Diabetes is Unpredictable - Diabetes Adds a Layer of Challenge to Daily Life - Struggle to Find Suitable Healthcare Providers |
| Diet and Nutrition Management | - Balanced Diet and Food Strategies - Role of Diabetes Education | Pregnancy and Diabetes | - Managing Pregnancy with Diabetes - Challenges of Pregnancy with Diabetes | Health Literacy in Diabetes Care | - Lack of Knowledge about Condition and Management - Struggle to Access Suitable Resources |

**Table S1**. Themes and subthemes identified by ChatGPT-3.5 during the first analysis round (left) and second analysis round (middle), and the human analyzer (right) from 81 codes.
